# High Productivity of Eicosapentaenoic Acid and Fucoxanthin by a Marine Diatom *Chaetoceros gracilis* in a Semi-Continuous Culture

**DOI:** 10.3389/fbioe.2020.602721

**Published:** 2020-12-11

**Authors:** Saki Tachihana, Norio Nagao, Tomoyo Katayama, Minamo Hirahara, Fatimah Md. Yusoff, Sanjoy Banerjee, Mohamed Shariff, Norio Kurosawa, Tatsuki Toda, Ken Furuya

**Affiliations:** ^1^Department of Science and Engineering for Sustainable Innovation, Faculty of Science and Engineering, Soka University, Tokyo, Japan; ^2^Laboratory of Marine Biotechnology, Institute of Bioscience, Universiti Putra Malaysia, Seri Kembangan, Malaysia; ^3^Department of Aquatic Bioscience, Graduate School of Agricultural and Life Sciences, The University of Tokyo, Tokyo, Japan; ^4^Department of Aquaculture, Universiti Putra Malaysia, Seri Kembangan, Malaysia; ^5^Faculty of Veterinary Medicine, Universiti Putra Malaysia, Seri Kembangan, Malaysia

**Keywords:** microalgae production, eicosapentaenoic acid, fucoxanthin, *Chaetoceros gracilis*, dilution rate

## Abstract

Significantly high eicosapentaenoic acid (EPA) and fucoxanthin contents with high production rate were achieved in semi continuous culture of marine diatom. Effects of dilution rate on the production of biomass and high value biocompounds such as EPA and fucoxanthin were evaluated in semi-continuous cultures of *Chaetoceros gracilis* under high light condition. Cellular dry weight increased at lower dilution rate and higher light intensity conditions, and cell size strongly affected EPA and fucoxanthin contents. The smaller microalgae cells showed significantly higher (*p* < 0.05) value of 17.1 mg g-dw^–1^ fucoxanthin and 41.5% EPA content per total fatty acid compared to those observed in the larger cells. *Chaetoceros gracilis* can accumulate relatively higher EPA and fucoxanthin than those reported previously. In addition, maintenance of small cell size by supplying sufficient nutrients and light energy can be the key for the increase production of valuable biocompounds in *C. gracilis*.

## Introduction

Microalgae have been utilized for the production of high value-added compounds such as carotenoids (Foo et al., 2015, 2017) and poly unsaturated fatty acids (PUFA) including eicosapentaenoic acid (EPA) and docosahexaenoic acid (DHA) (Chrismadha and Borowitzka, 1994; Yang et al., 2017). Among different microalgal groups, diatoms are known to be efficient producers of fucoxanthin and EPA, and have been widely used in aquaculture for feeding juvenile fish and shrimp to improve growth rate, survival rate and nutritional value (Enright et al., 1986). In addition to aquaculture, microalgae with high EPA and fucoxanthin contents have the potential to be used in food, cosmetic and pharmaceutical industries. Furthermore, EPA is one of the essential fatty acids which humans are unable to produce, and has both anti-arteriosclerosis (Hata et al., 1983) and anti-inflammation properties (Mickleborough et al., 2009). Fucoxanthin has anti-oxidation and anti-obesity effects on humans (Kanazawa, 2012). Therefore, there is a need for technological development to enhance valuable compounds production by diatoms to meet high social and economic demands (Krichnavaruk et al., 2007; Monkonsit et al., 2011; Kaspar et al., 2014).

Sufficient light intensity and nutrient supply are required for effective microalgal production. Since algal production is the process whereby light energy is transferred to chemical energy by photosynthesis (Mata et al., 2010), theoretically, high growth rate can be obtained under high light intensity. However, excess light can cause photoinhibition and decrease or impair the growth rate (Imaizumi et al., 2016). Therefore, light intensity should be adjusted to an optimal level, which is determined by the cell density.

Nutrient supply is also critical for effective biomass production. In high cell density culture with sufficient light intensity, nutrients are absorbed rapidly, thus resulting in inadequate nutrients for the cells. Light intensity and nutrient concentration affect not only biomass productivity, but also physiological cell quality. In high-light stress conditions, algal cells tend to lower cell division rate and store excess light energy in the form of starch and/or lipids (Williams and Laurens, 2010; Xin et al., 2010). On the other hand, insufficient nutrient concentration can also decrease biomass yield and alter the cell state. For example, Kaspar et al. (2014) observed that increase in the cell size and morphology transformation of *Chaetoceros calcitrans* cultured in batch mode occurred at the late phase of culture when nutrients were almost exhausted. This observation suggested that morphological change could be caused by nutrient deprivation. However, the relationship between the cell state and the production of biocompounds, such as EPA and fucoxanthin, has not been adequately investigated. Thus, it is necessary to investigate the mechanisms involved in the accumulation of these valuable compounds by a specific diatom at varying cell states under different light intensities and nutrient conditions.

The dilution rate in semi-continuous or continuous cultures can effectively regulate both light intensity and nutrient availability to the microalgal cells, which are the main operational factors for high biomass production, which in turn affect growth, cell composition and production of valuable compounds (Fernández Sevilla et al., 1998). At high dilution rate, high nutrient supply to algal cells can be achieved. However, cell density tends to decline with the high rate of periodical outflow of culture. A decrease in cell density causes an increase in light intensity exposure per cell, and accordingly cause photoinhibition when a photobioreactor receives high light energy. In contrast, at low dilution rate, photoinhibition is prevented for most of the culture period owing to increase of cell density. However, due to high cell density with low nutrient feed rate under sufficient light, rapid intake of nutrients by cells is likely to cause nutrient deficiency. Thus, dilution rate affects both light intensity and nutrient supply rate, and subsequently, affecting biomass production and chemical composition of the cell. Thus, by optimizing dilution rate, there is a potential for high biomass productivity and enhancing cells to contain intended valuable compound. In order to maximize biomass productivity and improve the concentration of EPA and fucoxanthin production by diatoms, optimization of dilution rate is necessary in a continuous culture under high light intensity. Therefore, the objective of this study was to examine the effect of dilution rate on biomass production and accumulation of EPA and fucoxanthin by the marine diatom *Chaetoceros gracilis* in semi-continuous culture under high irradiance.

## Materials and Methods

### Microalga and Culture Medium

*Chaetoceros gracilis* (UPMC-A0010-2) was isolated from Port Dickson, Malaysia and cultured in modified Conway medium which consisted of (per liter): 200 mg of KNO_3_; 20 mg of Na_3_PO_4_; 45 mg of Na_2_EDTA; 33.6 mg of H_3_BO_3_; 1.3 mg of FeCl_3_⋅6H_2_O; 0.36 mg of MnCl_2_⋅4H_2_O; 2.1 mg of ZnCl_2_; 2.0 mg of CoCl_2_⋅6H_2_O; 0.9 mg of (NH4)_6_Mo_7_O_24_⋅4H_2_O; 2.0 mg of CuSO_4_⋅5H_2_O; 0.2 mg of Thymine; 0.01 mg of Cyanocobalamin; and 15 mg of Na_2_SiO_3_⋅9H_2_O. The pre-culture was grown in a 1 L column reactor at 30°C under light intensity of 300 μmol m^–2^ s^–1^. The culture was aerated at a rate of 0.2 L min^–1^.

### Culture Conditions

Three different dilution rates of 0.1, 0.2, and 0.4 d^–1^ were applied to the *C. gracilis* culture. The temperature was maintained at 30 ± 1°C and 2% CO_2_ gas was added through a 0.2 μm filter at the flow rate of 0.2 L min^–1^. The volume of fresh medium supplied for dilution rates of 0.1, 0.2, and 0.4 d^–1^ was 100, 200, and 400 mL, respectively. Light intensity of 300 μmol m^–2^ s^–1^ was applied in all dilution rate treatments. In the dilution rates of 0.2 and 0.4 d^–1^, light intensity increased up to 1,000 μmol m^–2^ s^–1^. Since nutrient intake increased with the increase in light intensity, 2.5 times higher nutrient concentration than that under 300 μmol m^–2^ s^–1^ was added. The photoperiod was maintained at 12 L:12 D in all experimental conditions.

### Photobioreactor Setup

**FIGURE 1:**
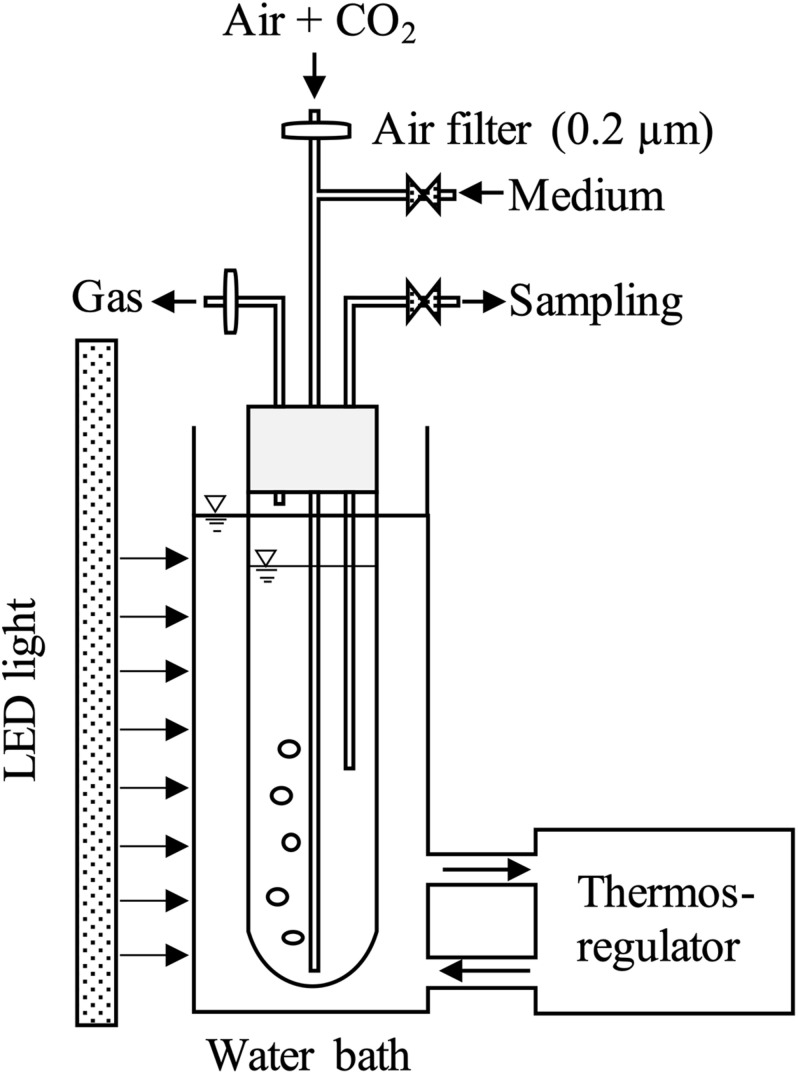
Schematic diagram of the column photobioreactor: inner diameter of 6.0 cm, working volume of 1.0 L. Light was provided to the surface of the water bath at 12L:12D. Filtered air (0.2 μm) was added with 2% CO_2_ from the bottom of the column reactor at a flow rate of 0.2 L min^–1^.

The culture system consisted of three parts: (1) a column reactor with culture medium supplying system, (2) a LED light, and (3) a thermos-regulator with a water bath (Figure 1). The column reactor was made of a transparent glass with an inner diameter of 6.0 cm and a height of 52 cm. Working volume and light-receiving area in this reactor were 1.0 L and 0.021 m^2^, respectively. The LED light was installed along the reactor. The light intensity was confirmed at the surface of the reactor using a quantum sensor (QSPL-2101, Biospherical Instruments, United States). Carbon dioxide concentration was controlled by a flow meter, and air with 2% CO_2_ at a rate of 0.2 L min^–1^ was introduced to the reactor through 0.2 μm filter continuously. The temperature was maintained by a thermos-regulator with a water bath. An electric fan was installed at the back side of LED light to dissipate heat. Sampling was conducted once in a day before the light period, and fresh medium was added until the culture volume reached 1.0 L. All operations were conducted under sterilized conditions.

### Analytical Methods

Dry weight, cell numbers, nutrient concentration, pigment composition and fatty acid composition were measured in triplicates. Cellular dry weight and areal production rates were calculated.

Biomass concentration was determined by dry weight (g-dw L^–1^) and cell number (cells mL^–1^) counts. For dry weight measurement, cell suspensions were filtered through Whatman GF/A filter and rinsed with 0.5 M ammonium formate to remove NaCl. The filters were dried in an oven at 70°C for 24 hrs and cooled down to room temperature in a desiccator before weighing. Cell numbers were counted daily using a hemocytometer. Cellular dry weight (CDW: ng-dw cell^–1^) was calculated using the following equation:

(1) $$\mathrm{CDW}=\frac{\mathrm{DW}}{N}$$

where DW is dry weight (g-dw L^–1^) and N is cell numbers (cells mL^–1^).

Samples for nutrient analyses (NO_3_-N, PO_4_-P, and SiO_2_-Si) were collected from filtrate through Whatman GF/A filters. The aliquots were stored at −20°C and thawed for analysis by an autoanalyzer (QuAAtro 39, BL TEC, Japan).

The dominant pigments for diatoms, chlorophyll *a* and fucoxanthin, were analyzed using a LCMS (liquid chromatography mass spectroscopy, Prominence-i LC-2030 3D, Shimazu Co., Ltd., Kyoto, Japan). Samples were filtered through glass fiber filters (GF/A, Whatman, United States) for the pigment analysis. The filters frozen at −80°C were thawed and soaked with 2 mL methanol, then extracted by ultrasound for 10 min. The samples were stored at 4°C for up to 72 h, and then the pigment analysis was conducted by using the LCMS equipped with a reversed phase column (XBridge, 2.1 × 150 mm i.d., C18, 5 μm, Waters Co., Ltd., Tokyo, Japan) and a photodiode array detector. Aliquots of 10 μL were used for the LCMS analysis. The mobile phase consisted of the eluent A (methanol: 0.5 M ammonium acetate = 8:2, v/v) and eluent B (methanol: ethyl acetate = 7:3, v/v) as described by Wright (1997). The following elution gradient was used: 0% B for 24.9 min, 100% B at 25 min for 9 min, 0% B until the end of the run at 39 min. The flow rate was maintained at 0.5 mL min^–1^, and the column temperature was 40°C during the analysis. The peaks were identified using standards obtained from the Danish Hydraulic Institute (Denmark).

For the extraction and analysis of fatty acids methyl ester (FAME), aliquots were filtered through a GF/A filter every 3 days and subsequently the filters were kept at −25°C until subjected to fatty acid analysis. The fatty acid composition was analyzed by the modified method of Bligh and Dyer (1959). The filtered samples were soaked in a 3 mL chloroform: methanol (1: 2, v/v) solution and extracted by sonication for 10 min. The extract was centrifuged at 2,000 rpm for 10 min, and the supernatant was collected. Ultrapure water of 10 mL was added to the extract, and then the extract was separated to remove impurities by centrifugation at 2,000 rpm for 10 min.

A known amount of C21 (heneicosane) was added to the extract as the internal standard. Fatty acids in the extract were transmethylated with acetyl chloride: methanol (5:100, v/v) solution at 100°C for 60 min to form FAME. The FAME was immersed in hexane and quantified by a gas chromatograph-mass spectrometer (GC-MS) (6890N GC/5973MS, Agilent Technologies, United States), equipped with a capillary column (30 m DB-5MS, Phenyl-Methyl/Silicone: J&W Scientific Inc., Folsom, CA, United States). Helium was used as a mobile phase at a flow rate of 1 mL min^–1^. The oven temperature was raised from 60 to 310°C at a rate of 5°C min^–1^. The injector and ion source temperatures were held at 310 and 230°C, respectively. Data analysis software (Agilent MSD Productivity ChemStation For GC and GC-MS Systems Data Analysis, Agilent Technologies, United States) was used for GC-MS data analysis. Identification of each fatty acid was conducted by comparison between the retention time and the mass spectrum of the standard, and quantified by comparing their peak area with that of the internal standard.

Areal production rate (P_*A*_: g-dw m^–2^d^–1^) was calculated according to the following equation:

(2) $$P_{A}=\frac{{\mathrm{DW}}_{n}\times V-{\mathrm{DW}}_{n-1}\times V\times \left(1-R\right)}{A\times T}$$

Where DW_*n*_ is dry weight at day *N* (g-dw L^–1^), V is volume of the reactor (L), R is dilution rate (d^–1^), A is surface area of the reactor (m^2^), and T is culturing time (day).

### Statistical Analysis

All data were tested for normality before being analyzed using a two-way ANOVA to assess the impacts of dilution rates, light intensity and their interactions at *p* < 0.05.

## Results and Discussion

### Cell Density and Cellular Dry Weight

**FIGURE 2:**
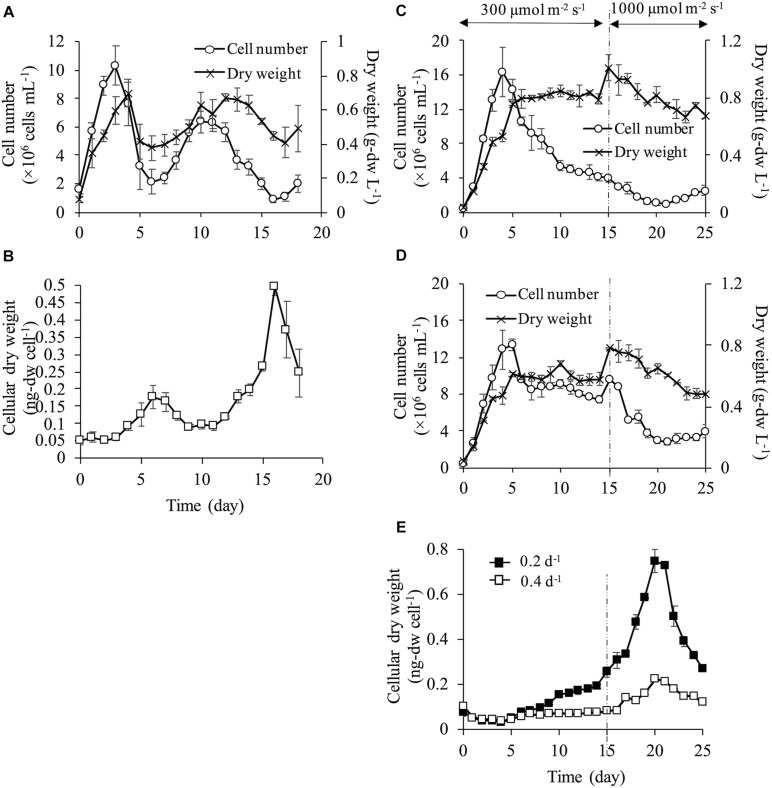
Growth characteristics at each dilution rate. Cell number and dry weight **(A)** and cellular dry weight **(B)** at dilution rate of 0.1 d^–1^ under 300 μmol m^–2^ s^–1^. At dilution rates of 0.2 and 0.4 d^–1^, light intensity was set at 300 μmol m^–2^ s^–1^ during days 0–14, and at 1,000 μmol m^–2^ s^–1^ during days 15–25. Cell number and dry weight at dilution rate of 0.2 d^–1^ **(C)** and dilution rate of 0.4 d^–1^ **(D)**. Cellular dry weight of dilution rates of 0.2 d^–1^ and 0.4 d^–1^ **(E)**.

Cell density fluctuated and did not reach a steady state at the dilution rate of 0.1 d^–1^ (Figure 2A). The cell numbers on day 0 was 1.56 ± 0.26 × 10^6^ cells mL^–1^, and then sharply increased up to 10.3 ± 1.3 × 10^6^ cells mL^–1^ on day 3. However, the cells decreased to 2.15 ± 0.87 × 10^6^ cells mL^–1^ on day 6 and fluctuated subsequently. Dry weight exhibited a similar trend of fluctuation as the cell numbers, but the was not exactly in the same proportion. The difference in the proportion of the change between the cell numbers and dry weight indicated that the dry weight per single cell changed during the experimental period. Cellular dry weight was approximately 0.05 ng-dw cell^–1^ until day 4 when the cell numbers reached the maximum values (Figure 2B). However, cellular dry weight increased up to 0.180 ng-dw cell^–1^ on day 7 with the decrease in cell numbers. During day 7–11, when the second increase in cell numbers occurred, cellular dry weight decreased again. On day 16 when cell numbers reached the minimum, cellular dry weight increased up to the maximum of 0.50 ng-dw cell^–1^ which corresponded to the 10-fold increase of cellular dry weight at the beginning of the culture. The dilution rate of 0.1 d^–1^, which provided the lowest nutrient supply and the lowest discharge of culture medium did not show a steady state of cell density and cellular dry weight in a semi-continuous culture. This result showed that cell physiological condition changed possibly due to insufficient nutrient or other stress factors, and thus stable cell division rate could not be maintained. The steady state of both cell density and cell physiology could not be attained at a dilution rate of 0.1 d^–1^ suggesting that both consistent cell quality and stable biomass production rate could not be achieved in the actual production process for commercialization.

At a dilution rate of 0.2 d^–1^, dry weight increased until day 5 and it reached a steady state at approximately 0.8 g L^–1^ during the light intensity of 300 μmol m^–2^ s^–1^. However, the cell numbers showed different behavior from the dry weight under the same light condition. The cell numbers increased rapidly from the onset of the culture and reached the maximum of 16.4 ± 2.9 × 10^6^ cells mL^–1^ on day 4. Then, the cell numbers continued decreasing after day 4, although the dry weight was maintained (Figure 2C). After increasing the light intensity up to 1,000 μmol m^–2^ s^–1^, the cell numbers continued decreasing and dry weight surpassed 0.8 g L^–1^ for a few days. However, dry weight gradually decreased after that and was stable at a lower level compared to values under 300 μmol m^–2^ s^–1^. This result showed that a stable dry weight under 300 μmol m^–2^ s^–1^ could be achieved due to the increase in cellular dry weight, suggesting that cell physiological condition could be changed in the process.

**FIGURE 3:**
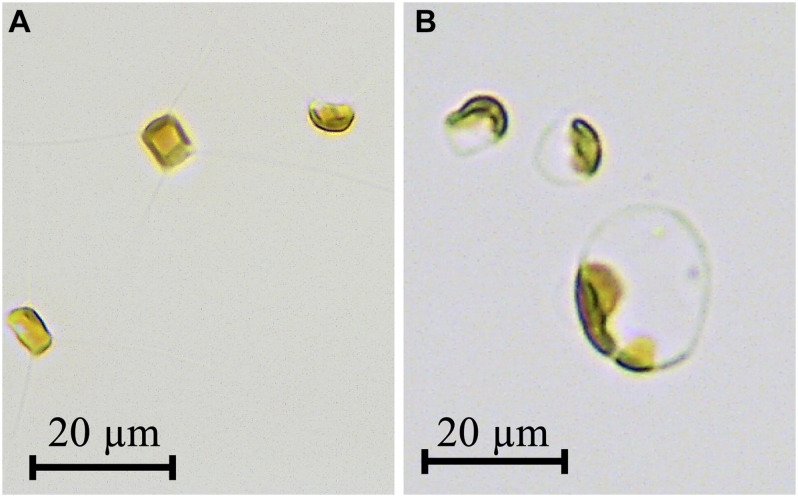
Photomicrographs of *Chaetoceros gracilis* grown at dilution rate of 0.2 d^–1^ under 300 μmol m^–2^ s^–1^ **(A)** and dilution rate of 0.2 d^–1^ under 1,000 μmol m^–2^ s^–1^ **(B)**. Scale bar = 20 μm.

During the late phase of the culture at a dilution rate of 0.2 d^–1^, unusually large cells of about 20 μm in diameter were observed (Figure 3B). Compared to cells in the initial phase (Figure 3A), their appearance changed from typical rectangular to round-shape with an increase in cell size. Furthermore, a brown plastid only occupied up to a small portion of the large round cell, although the plastid completely filled up the small rectangular cell. This indicated that pigment contents per dry weight decreased in large round-shaped cells.

At a dilution rate of 0.4 d^–1^ under 300 μmol m^–2^ s^–1^, both the cell density and dry weight reached a steady state after day 6 (Figure 2D) and cellular the dry weight was stable at low values of <0.1 ng-dw cell^–1^ (Figure 2E). Increases in cell numbers and dry weight were observed temporally after day 15 with increasing light intensity but cell numbers decreased after a few days. While only dry weight reached a steady state at a dilution rate of 0.2 d^–1^ under 300 μmol m^–2^ s^–1^, both cell numbers and dry weight were maintained at a dilution rate of 0.4 d^–1^. Cellular dry weight at a dilution rate of 0.4 d^–1^ was maintained by small-sized cells ranging from 0.04 to 0.07 ng-dw cell^–1^ under a light intensity of 300 μmol m^–2^ s^–1^. However, the cell size increased resulting in a maximum cellular dry weight of 0.22 ng-dw cell^–1^ on day 20 under 1,000 μmol m^–2^ s^–1^ even at the highest dilution rate (i.e., the highest nutrient supplying rate). At dilution rates of 0.2 and 0.4 d^–1^, the cell numbers slightly increased from day 20 and cellular dry weight decreased under a light intensity of 1,000 μmol m^–2^ s^–1^. This result indicated that cell division started again from day 20 as seen by the smaller cell size (Figure 2E).

Generally, in semi-continuous and continuous cultures, nutrients are introduced in a reactor periodically or continuously, and microalgae use the nutrients for their growth. Therefore, biomass production increases with increasing nutrient availability if sufficient light energy and carbon dioxide are provided (Ruiz-marin et al., 2010; Mcginn et al., 2012; Imaizumi et al., 2014). However, interestingly this general phenomenon was not observed in the current study. It was illustrated that under a relatively high light intensity of 300 μmol m^–2^ s^–1^, *C. gracilis* did not maintain its cell numbers and dry weight at a low dilution rate of 0.1 and 0.2 d^–1^ even under continuous nutrient supply. The phenomenon of increase in cellular dry weight was observed (1) at lower dilution rate, which was low flow rate of culture media and (2) at higher light intensity, where cells consumed high amount of nutrient to grow. This suggested that stress factors such as nutrient deprivation, accumulation of inhibitor in the medium and excess light intensity would attribute to the decrease in cell numbers and enlargement of cell size.

### Nutrient Concentration

**FIGURE 4:**
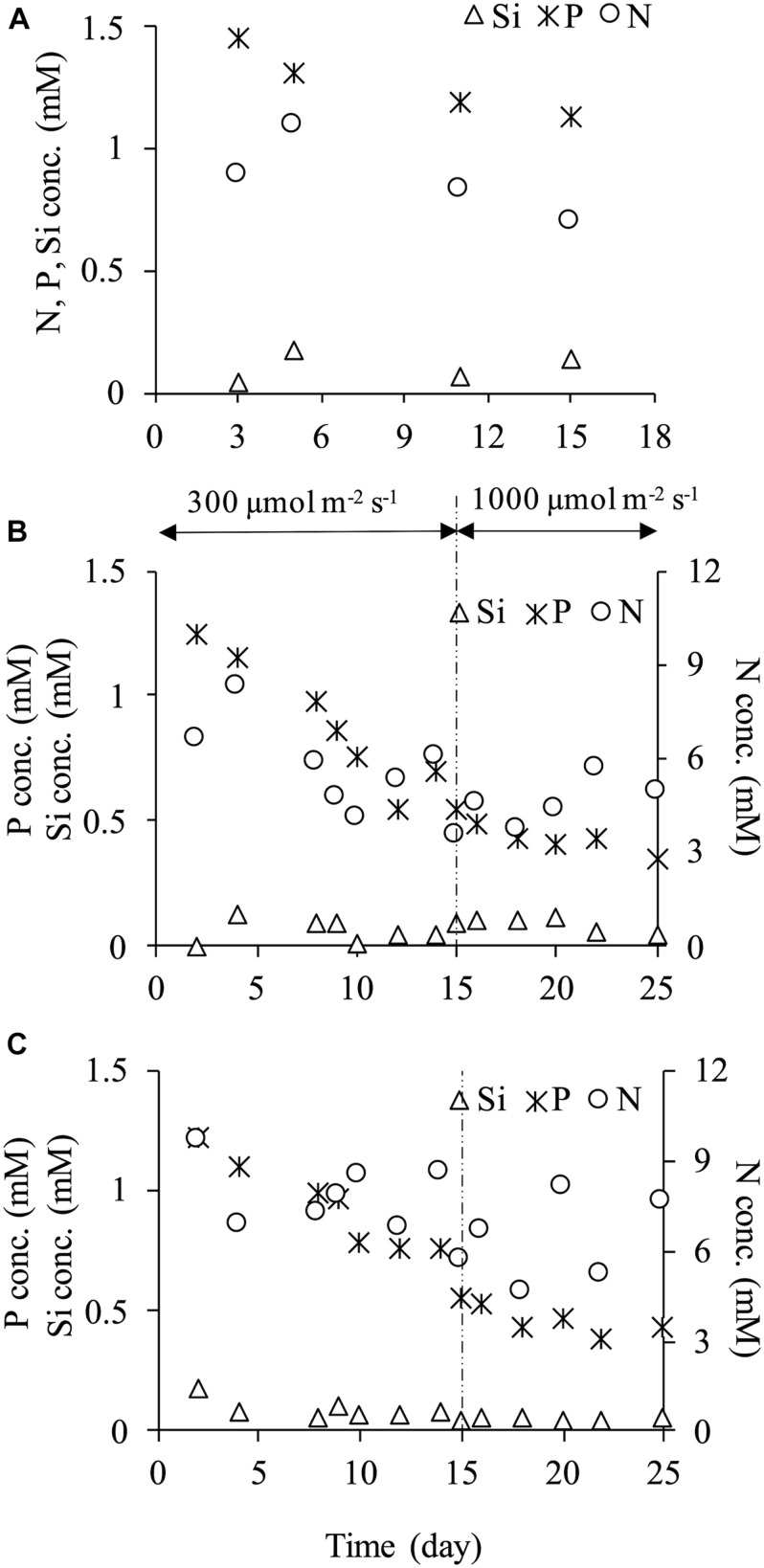
Nutrients concentration (NO_3_-N, PO_4_-P, SiO_2_-Si) at dilution rate of 0.1 d^–1^ **(A)**, 0.2 d^–1^ **(B)** and 0.4 d^–1^ **(C)**.

Concentrations of nitrogen (NO_3_-N), phosphorus (PO_4_-P) and silicon (SiO_2_-Si) in the culture media showed their decreased concentrations with time indicating their uptake by the microalgae (Figure 4). At every dilution rate, nitrogen and phosphorus decreased during the culture, yet they did not become depleted. In the case of high light intensity of 1,000 μmol m^–2^ s^–1^ at dilution rates of 0.2 and 0.4 d^–1^, nitrogen and phosphorus concentrations were at least more than 3.60 and 0.35 mM, respectively. In contrast, the silicon concentration was significantly low during the culture period. The ratio of silicon concentration in the reactor to an input concentration of 1.88 mM was low, and 95.5 and 96.3% of silicon were absorbed at dilution rates of 0.2 and 0.4 d^–1^, respectively. A considerable increase in cellular dry weight was observed in a dilution rate of 0.2 d^–1^ under a light intensity of 1,000 μmol m^–2^ s^–1^ as mentioned above, and this phenomenon was possibly attributed to the depletion of silicon. Kaspar et al. (2014) reported that change in cell morphology and enlargement of cell size of *C. calcitrans* could be caused by nutrient depletion. In the current study, more silicon supply might be necessary to avoid the cell enlargement.

The high silicon uptake was also observed even at the dilution rate of 0.4 d^–1^ under 300 μmol m^–2^ s^–1^ (Figure 4C). Tantanasarit et al. (2013) examined nutrient uptake kinetics by *C. calcitrans* in high nutrient concentrations, and they showed that this species could absorb nutrient beyond its requirement level and accumulated it in its vacuoles. In *C. gracilis* cells cultured in the present study, a similar phenomenon may have occurred. In other words, *C. gracilis* possibly absorbed more silicon than was needed and accumulated it in its vacuoles. Cell enlargement might have occurred due to silicon deficiency as the diatom has a high demand for silicon, resulting in unsustainable growth.

### Production Rate

**FIGURE 5:**
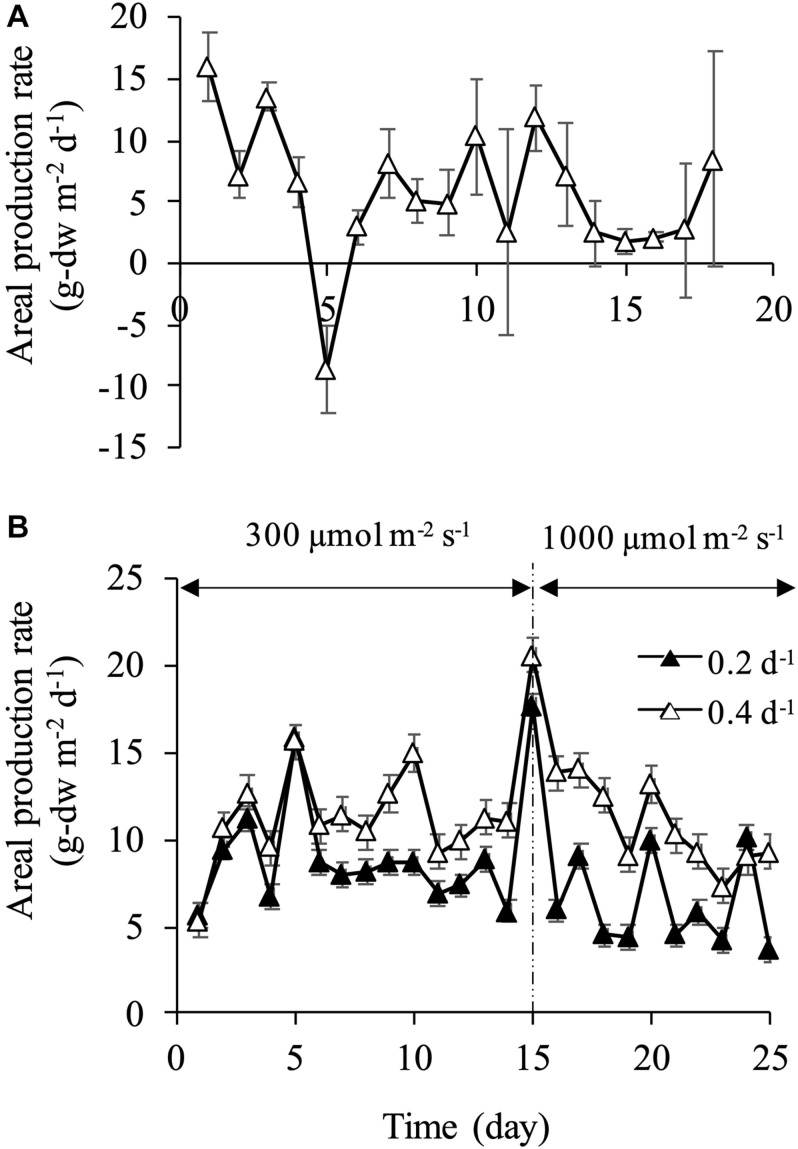
Areal and volumetric production rates at dilution rate of 0.1 d^–1^ **(A)** and dilution rates of 0.2 and 0.4 d^–1^** (B)**.

At a dilution rate of 0.1 d^–1^, areal production rate became negative value on day 5 due to the fluctuation of cell density and dry weight (Figure 5A). Overall, aerial production rate varied significantly in the range of −8.61 to 16.1 g-dw m^–2^ d^–1^. The relatively stable production rate was attained at dilution rates of 0.2 and 0.4 d^–1^, which ranged from 7 to 9 g-dw m^–2^ d^–1^ and from 10 to 14 g-dw m^–2^ d^–1^, respectively (Figure 5B). The production rate increased instantaneously on day 15 when light intensity was increased to 1,000 μmol m^–2^ s^–1^, and the maximum production rate of 17.7 g-dw m^–2^ d^–1^ at a dilution rate of 0.2 d^–1^ and 20.6 g-dw m^–2^ d^–1^ at a dilution rate of 0.4 d^–1^ were observed, corresponding to volumetric production rate of 0.372 and 0.433 g-dw L^–2^ d^–1^, respectively. In previous studies of biomass production by diatoms, areal production of 2.24 g-dw m^–2^ d^–1^ by *C. gracilis* (Hatate et al., 1998), 10.1 g-dw m^–2^ d^–1^ by *Nitzschia* sp. (Silva-Aciares and Riquelme, 2008), 13.1 g-dw m^–2^ d^–1^ by *Fistulifera* sp. (Satoh et al., 2013), and 15.4 g-dw m^–2^ d^–1^ by *C. muelleri* (Zhang and Richmond, 2003) were reported. In the current study, similar results of 15–20 g-dw m^–2^ d^–1^ were observed at the dilution rate of 0.4 d^–1^. However, production rate decreased after a few days under 1,000 μmol m^–2^ s^–1^. This result might be attributed to excessive light intensity and/or silicon limitation. After day 15, areal production rates at both dilution rates of 0.2 and 0.4 d^–1^ decreased, but were maintained at approximately 6 and 10 g-dw m^–2^ d^–1^, respectively. At a dilution rate of 0.2 d^–1^ under 300 μmol m^–2^ s^–1^, relatively high stable production rate was obtained due to an increase in dry weight with enlargement of the cell. In other words, the cell physiological condition was not stable at the dilution rate of 0.2 d^–1^ under 300 μmol m^–2^ s^–1^, although the production rate reached a steady state. These results showed that the biomass production rate in terms of dry weight could be maintained at relatively high values in both dilution rates of 0.2 and 0.4 d^–1^. However, the stable production of a uniform-size cell was achieved only in the condition of 0.2 d^–1^ under 300 μmol m^–2^ s^–1^. Therefore, appropriate nutrient supply and optimum light intensity are crucial to secure homogeneity of products and high productivity.

### Fatty Acid and Pigment Composition

Cellular content of valuable compounds is quite important in the process of maximizing microalgae productivity. Fatty acid composition (as% total fatty acid) on day 4 showed the minimum cellular dry weight and on day 20 the maximum as shown in Table 1. Fatty acids in *C. gracilis* mainly consisted of C16, C16:1n-7, C16:3, C20:5n−3. According to the comparison of fatty acid composition in small cells on day 4 and in large cells on day 20, saturated fatty acid (SFA) contents in small cells at both dilution rates showed significantly (*p* < 0.05) lower percentages of 14.6 and 18.3%, respectively, compared to those of large cells. Monounsaturated fatty acid (MUFA) contents in the small cells were also relatively low. Conversely, polyunsaturated fatty acid (PUFA) content including EPA showed significantly higher values of 60 and 55%, respectively, associated with small cells on day 4, compared to the large cells.

**TABLE 1:** Fatty acid composition (as % of total fatty acid) of *C. gracilis* on days 4 and 20 at dilution rates of 0.2 and 0.4 d^–1^.

	*C. gracilis*				*C. gracilis*		*C. gracilis*	*T. pseudonana*	*C. sorokiniana*
	Semi-continuous				Batch		Batch	Batch	Batch
	0.2 d^–1^		0.4 d^–1^		–	–	–	–	–
						
	Day 4	Day 20	Day 4	Day 20	Day 3	Day 10			
C14	3.3	11.2	5.9	9.2	15.9	13.0	8.8	14.3	0.1
C15	0.4	0.8	0.5	0.5	0.9	0.9	1.0	0.8	–
C16	10.3	30.7	11.3	23.7	35.8	24.0	23.3	11.2	21.4
C17	–	–	–	–	–	–	0.3	0.1	–
C18	0.7	1.3	0.6	0.9	–	3.2	4.1	0.7	1.2
Other	–	–	–	–	7.0	2.5	1.2	0.1	0.8
ΣSFA	14.6	43.9	18.3	34.3	59.6	43.6	38.7	27.2	23.5
C14:1	–	–	–	–	–	0.7	–	–	–
C16:1	–	–	–	–	–	–	–	–	3.9
C16:1n−9	–	–	–	–	14.4	–	–	–	–
C16:1n−7	25.4	36.9	25.9	37.5	–	–	33.4	18.0	–
C18:1	–	–	–	–	–	–	–	–	32.8
C18:1n−11	–	–	–	–	–	7.5	–	–	–
C18:1n−9	–	0.5	0.7	0.4	20.9	17.4	3.6	0.5	–
C18:1n−7	–	–	–	–	–	–	1.7	0.1	–
Other	–	–	–	–	0.7	5.2	1.3	0.9	0.9
ΣMUFA	25.4	37.4	26.6	38.0	36.0	30.8	40.0	19.5	37.6
C16:2	–	–	–	–	0.5	0.9	–	–	1.5
C16:3	16.1	5.8	17.6	1.3	–	–	–	–	–
C16:3n−4	–	–	–	–	–	–	2.3	12.7	–
C18:2	–	–	–	–	0.7	–	–	–	30.3
C18:2n−9	–	–	–	–	–	–	2.0	–	–
C18:2n−6	–	2.4	1.5	2.4	3.2	0.6	0.5	0.4	–
C18:3	–	–	–	–	–	–	–	–	5.7
C20:2n−6	–	–	–	–	–	21.5	–	–	–
C20:4n−6	2.4	1.9	1.4	16.7	–	0.4	4.5	0.3	–
C20:5n−3	41.5	8.6	34.6	27.8	–	–	4.6	19.3	–
C22:6n−3	–	–	–	–	–	–	0.3	3.9	–
Other	–	–	–	–	–	2.2	5.6	16.0	0.4
ΣPUFA	60.0	18.7	55.0	7.3	4.4	25.6	19.8	52.6	37.9
References	This study				Pratiwi et al., 2009	Pratiwi et al., 2009	Volkman et al., 1989	Volkman et al., 1989	Li et al., 2013

**FIGURE 6:**
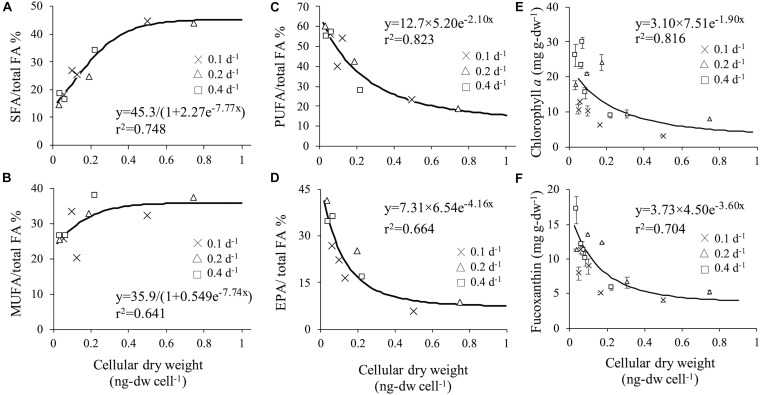
Correlation between cellular dry weight and SFA **(A)**, MUFA **(B)**, PUFA **(C)** and EPA **(D)**. Correlation between cellular dry weight and chlorophyll *a* **(E)** and fucoxanthin **(F)**.

Relationship between cellular dry weight and SFA, MUFA, PUFA, and EPA contents per total fatty acid were evaluated because of the difference between fatty acid composition in different cell sizes (Figures 6A–D). Significant positive correlation of SFA (*y* = 45.3/(1 + 2.27 e^–7.77x^), *r*^2^ = 0.748) and MUFA (*y* = 35.9/(1 + 0.549 e^–7.74x^), *r*^2^ = 0.641) to cellular dry weight was obtained. On contrary, significant negative correlation of PUFA (*y* = 12.7 × 5.20 e^–2.10x^, *r*^2^ = 0.823) showed that bigger cell size accumulated less PUFA content.

In previous studies using *C. gracilis*, SFA and MUFA in total was 78.7% (Volkman et al., 1989) and 74.4% on day 10 in batch culture (Pratiwi et al., 2009) which were similar to the data on day 20 of the present study. According to Pratiwi et al. (2009), cells on day 3 marked high SFA content of 95.6% and low PUFA content of 4.4%. Volkman et al. (1989) reported a high PUFA content of 52.6% in *Thalassiosira pseudonana.* EPA content was previously reported with 5.0% (Tokushima et al., 2016) and 5.7% (Volkman et al., 1989) in *C. gacilis*. and 10.94% (Foo et al., 2017 and 20.8% (Natrah et al., 2007) in *C. calcitrans* (Table 2). Wu et al. (2016) reported EPA content of 25.65% in *Phaeodactylum tricornutum*. In the present study, high PUFA content of 60% and significant high EPA contents of 41.5% in total fatty acid was associated with small cells of *C. gracilis*. These results indicated that cell size affects PUFA, mainly EPA, indicating that maintenance of small cellular dry weight is the key operation factor for effective production of EPA.

**TABLE 2:** Biomass productivity, contents and productivities of fucoxanthin and EPA.

Algae	Type of reactor	Culture mode	Biomass		Fucoxanthin			EPA				References
						
			Volumetric productivity	Areal productivity	Content	Volumetric productivity	Areal productivity	Composition	Content	Volumetric productivity	Areal productivity	
			(g-dw L^–^^1^ d^–^^1^)	(g-dw m^–^^2^ d^–^^1^)	(mg g-dw^–^^1^)	(mg-dw L^–^^1^ d^–^^1^)	(mg-dw m^–^^2^ d^–^^1^)	(%TFA)	(mg g-dw^–^^1^)	(mg-dw L^–^^1^ d^–^^1^)	(mg-dw m^–^^2^ d^–^^1^)	
*Isochrysis galbana* (2 strains)	Bubble column	Batch	–	–	6.04-18.23	–	–	–	–	–	–	Kim et al., 2012
*Chaetoceros gracilis*	Bubble column	Batch	–	–	2.24	–	–	–	–	–	–	
*Phaeodactylum tricornutum*	Bubble column	Batch	–	–	8.55	–	–	–	–	–	–	
*Nitzschia* sp.	Bubble column	Batch	–	–	4.92	–	–	–	–	–	–	
*Chaetoceros calcitrans*	Bubble column	Batch	–	–	5.13	–	–	10.94	–	–	–	Foo et al., 2017
*Isochrysis galbana*	Bubble column	Batch	–	–	2.19	–	–	0.77	–	–	–	
*Skeletonema costatum*	Bubble column	Batch	–	–	0.36	–	–	4.19	–	–	–	
*Odontella sinensis*	Bubble column	Batch	–	–	1.18	–	–	10.46	–	–	–	
*Phaeodactylum tricornutum*	Bubble column	Batch	–	–	0.07	–	–	19.12	–	–	–	
*Isochrysis galbana* (14 strains)	Bubble column	Semi-cont. (*d* = 0.375 d^–1^)	0.14-0.48	2.08-10.02	8.9-15.8	1.07-5.85	25.2-137.8	–	–	–	–	Sun et al., 2019
*Odontella aurita*	Bubble column	Batch	0.34-0.56	9.4-15.0	4.28-18.14	1.82-7.96	42.9-187.4*	–	–	–	–	Xia et al., 2013
*Phaeodactylum tricornutum*	Flat plate	Semi-cont. (*d* = 0.03 d^–1^)	0.018-0.054	0.75-2.28	10.1-59.2	0.44-2.16	22.0-108.0	–	–	–	–	McClure et al., 2018
*Phaeodactylum tricornutum*	Flat plate	Batch	–	–	8	4.73	189*	15	–	9.58	383*	Gao et al., 2017
*Isochrysis* sp.	Flat plate	Semi-cont. (*d* = 0.4 d^–1^)	–	–	17	–	–	–	–	–	–	Crupi et al., 2013
*Navicula* sp.	Flat plate	Batch	0.56	–	–	–	–	8	56	56	–	Satoh et al., 2013
*Phaeodactylum tricornutum*	Pond	Semi-cont. (*d* = 0.2-0.4 d^–1^)	–	–	–	–	–	–	–	–	0.15	Veloso et al., 1991
*Cylindrotheca closterium*	Tubular	Cont.	–	–	5.34	–	–	–	–	–	–	Pasquet et al., 2011
*Chaetoceros gracilis*	Tube	Batch	–	–	1.0-5.2	0.04-0.68	0.1-1.6	2.8-5.0	–	–	–	Tokushima et al., 2016
*Phaeodactylum tricornutum*	Tube	Batch	–	–	–	–	–	29.8	62.3	–	–	Yongmanitchai and Ward, 1991
*Phaeodactylum tricornutum*	Flask	Batch	1.52	–	–	–	–	–	2.83	43.13	–	Cerón García et al., 2005
*Phaeodactylum tricornutum* (6 strains)	Flask	Batch	0.022-0.031	–	2.14-5.5	0.047-0.17	–	12.43-25.65		–	–	Wu et al., 2016
*Cyclotella cryptica*	Flask	Batch	0.18-0.25	–	6.5-12.9	1.2-3.4	–	–	–	–	–	Guo et al., 2016
*Chrysotila carterae*	Flask	Batch	–	–	1.04	0.024	–	–	–	–	–	Ishika et al., 2017
*Chaetoceros muelleri*	Flask	Batch	0.018	–	2.92	0.072	–	–	–	–	–	
*Phaeodactylum tricornutum*	Flask	Batch	0.022	–	1.87	0.041	–	–	–	–	–	
*Tisochrysis lutea*	Flask	Batch	0.027	–	2.05	0.055	–	–	–	–	–	
*Navicula* sp.	Flask	Batch	0.037	–	1.49	0.054	–	–	–	–	–	
*Amphora* sp.	Flask	Batch	0.047	–	1.21	0.053	–	–	–	–	–	
*Chaetoceros muelleri*	–	Batch	–	–	–	–	–	–	10.4	–	–	Martínez-Fernández et al., 2006
*Chaetoceros* sp.	–	Batch	–	–	–	–	–	–	15.4	–	–	
*Pavlova* sp.	–	Batch	–	–	–	–	–	–	30.8	–	–	
*Micromonas pusilla*	–	Batch	–	–	–	–	–	–	35.2	–	–	
*Chaetoceros calcitrans*	Flask	Batch	–	–	–	–	–	11.1	–	–	–	Volkman et al. (1989)
*Chaetoceros gracilis* (2 strains)	Flask	Batch	–	–	–	–	–	4.6-5.7	–	–	–	
*Skeletonema costatum*	Flask	Batch	–	–	–	–	–	6	–	–	–	
*Thalassiosira pseudonana*	Flask	Batch	–	–	–	–	–	19.3	–	–	–	
*Pavlova lutheri*	Flask	Batch	–	–	–	–	–	19.7	–	–	–	
*Chaetoceros calcitrans*	Bottle	Batch	–	–	–	–	–	20.8	–	–	–	Natrah et al., 2007
*Isochrysis galbana*	Bottle	Batch	–	–	–	–	–	8.4	–	–	–	
*Chaetoceros gracilis*	Bubble column	Semi-cont. (*d* = 0.1 d^–1^)	0.082	3.89	7.36	0.60	28.6	17.8	8.03	0.66	31.2	This study
*Chaetoceros gracilis*	Bubble column	Semi-cont. (*d* = 0.2 d^–1^)	0.168	7.99	11.8	1.98	94.3	25.2	11.07	1.86	88.5	
*Chaetoceros gracilis*	Bubble column	Semi-cont. (*d* = 0.4 d^–1^)	0.248	11.8	15.4	3.82	182	29.2	14.11	3.50	167	

*Xia et al. (2013) and Gao et al. (2017) conducted batch culture under continuous illumination condition. Cont., continuous culture; Semi-cont., semicontinuous culture; d, dilution rate; TFA, total fatty acid. The values of this study indicate the averages during the cultivation.

Microscopic observation revealed that changes in dilution and light intensity influenced both cell morphology and the amount of plastid (Figures 3A,B). Therefore, chlorophyll *a* which is the prime light harvesting pigment for photosynthesis and fucoxanthin which is utilized as valuable compound, were selected to examine for their relationship between cellular dry weight and each related pigment. High correlation with cellular dry weight was observed in chlorophyll *a* (*y* = 3.10 × 7.51e^–1.90x^, *r*^2^ = 0.816) and fucoxanthin (*y* = 3.73 × 4.50 e^–3.60x^, *r*^2^ = 0.704) (Figures 6E,F). Pigment per dry weight decreased as cellular dry weight increased. The maximum chlorophyll *a* content of 29.7 mg g-dw^–1^ was obtained at cellular dry weight of 0.071 ng-dw cell^–1^, and the maximum fucoxanthin content of 17.1 mg g-dw^–1^ at cellular dry weight of 0.037 ng-dw cell^–1^. It was illustrated that a small cell can accumulate approximately 3.76-fold chlorophyll *a* and 3.30-fold fucoxanthin compared to enlarged cells. Dilution rate represents the amount of nutrient supply, i.e., nutrient input at a dilution rate of 0.4 d^–1^ is four times larger than that at a dilution rate of 0.1 d^–1^. The fucoxanthin productivity at a dilution rate of 0.2 d^–1^ was half of that at a dilution rate of 0.4 d^–1^. This indicated that productivity proportionally changed with the input of nutrients. However, the productivity at a dilution rate of 0.1 d^–1^ was lower than a quarter of that at a dilution rate of 0.4 d^–1^, possibly due to the unstable cell physiological condition under insufficient nutrients. According to Volkman et al. (1989), *C. calcitrans* showed a tendency to contain higher chlorophyll *a* per unit volume in the smaller cell. Although no specific chlorophyll *a* content was stated in their study, the result obtained were similar to the present study.

Fucoxanthin is one of major carotenoid pigments found only in diatoms and brown algae. In previous studies, fucoxanthin contents of 8.55 mg g-dw^–1^ in *Phaeodactylum tricornutum*, 4.92 mg g-dw^–1^ in *Nitzschia* sp. (Kim et al., 2012) and 5.5 mg g-dw^–1^ in *P. tricornutum* were reported (Wu et al., 2016; Table 2). The present result was comparable to the values of 18.14 mg g-dw^–1^ reported by Xia et al. (2013) with *Odontella aurita* and 15.8-18.23 mg g-dw^–1^, obtained with *Isochrysis galbana* by Foo et al. (2017) and Sun et al. (2019). McClure et al. (2018) reported a greater content of 59.2 mg g-dw^–1^ for *P. tricornutum*, however, the areal fucoxanthin productivity is markedly lower (108.0 mg-dw m^–2^ d^–1^) due to their low biomass production rate. High yields of 189 mg-dw m^–2^ d^–1^ for *P. tricornutum* (Gao et al., 2017) and 187.4 mg-dw m^–2^ d^–1^ for *O. aurita* (Xia et al., 2013) were reported, however, both of these studies conducted in batch culture under continuous illumination condition. Meanwhile, the present study operated in semi-continuous culture under 12 h:12 h light-dark cycle. Many previous studies reported fucoxanthin contents in microalgae were conducted in a batch culture which was not supplied with additional nutrient during the culturing process. This study revealed that sufficient nutrient supply, especially silicon is one of the key operational factors to maintain small cells and to gain high fucoxanthin content of *C. gracilis*. These results raised the possibility that the fucoxanthin contents can be improved in other diatom species by maintaining the small-sized cells in a continuous culture with sufficient nutrients.

Furthermore, macroalgae also contain fucoxanthin which has high commercial value potential as a bioresource. Previous studies reported fucoxanthin contents of 4.49 ± 0.60 mg g-dw^–1^ in *Sargassum horneri* (Nomura et al., 2013), 0.2674 mg g-dw^–1^ in *Padina australis*, 0.2134 mg g-dw^–1^ in *Turbinaria conoides* (Zailanie and Purnomo, 2011) and 0.0297 mg g-dw^–1^ in *Laminaria* sp. (Frecha-ferreiro, 2010). Results obtained in the present study showed relatively higher fucoxanthin content of >10 mg g-dw^–1^ and high EPA contents compared to other macro- and microalgae reported. Therefore, *C. gracilis* can be regarded as a highly prospective species for production of EPA and fucoxanthin by optimizing operation with sufficient nutrient supply and appropriate light intensity.

## Conclusion

High biomass production, EPA and Fucoxanthin contents were achieved in semi-continuous culture of marine diatom, *C. gracilis.* At low dilution rate of 0.1 and 0.2, the cell weight increased and both EPA and fucoxanthin contents significantly decreased due to deficiency of nutrients, especially silicon. In order to obtain high biomass production and valuable biocompounds contents, it is important to keep cells small by providing sufficient nutrients for *C. gracilis*. Further studies should focus on perfusion culture which provides sufficient nutrients to further increase biomass and valuable biocompounds production in diatom culture.

## Data Availability Statement

The original contributions presented in the study are included in the article/supplementary material, further inquiries can be directed to the corresponding author.

## Author Contributions

ST contributed to the collection and analyzing of the data and the writing of the manuscript. NN, TK, and KF contributed to the conception and design of experimental work, supervised the project, reviewed, and edited the draft prior to the submission. SB and MS contributed to the writing of the manuscript. FY, KF, and TT contributed to the funding acquisition of the research. MH and NK contributed to the analysis of the samples and the interpretation of the data. All authors read and approved the submitted manuscript.

## Conflict of Interest

The authors declare that the research was conducted in the absence of any commercial or financial relationships that could be construed as a potential conflict of interest.
